# Acclimatization across space and time in the effects of temperature on mortality: a time-series analysis

**DOI:** 10.1186/1476-069X-13-89

**Published:** 2014-10-28

**Authors:** Mihye Lee, Francesco Nordio, Antonella Zanobetti, Patrick Kinney, Robert Vautard, Joel Schwartz

**Affiliations:** Department of Environmental Health, Exposure, Epidemiology, and Risk Program, Harvard School of Public Health, 401 Park Drive, Landmark Center West 4th fl, Boston, MA 02215 USA; Department of Environmental Health Sciences, Mailman School of Public Health at Columbia University, New York, NY USA; LSCE/IPSL, laboratoire CEA/CNRS/UVSQ, Orme des merisiers, 91191 Gif sur Yvette, Cedex, France

**Keywords:** Temperature and mortality, Acclimation, Acclimatization, Climate change, Global warming

## Abstract

**Background:**

Climate change has increased the days of unseasonal temperature. Although many studies have examined the association between temperature and mortality, few have examined the timing of exposure where whether this association varies depending on the exposure month even at the same temperature. Therefore, we investigated monthly differences in the effects of temperature on mortality in a study comprising a wide range of weather and years, and we also investigated heterogeneity among regions.

**Methods:**

We analyzed 38,005,616 deaths from 148 cities in the U.S. from 1973 through 2006. We fit city specific Poisson regressions to examine the effect of temperature on mortality separately for each month of the year, using penalized splines. We used cluster analysis to group cities with similar weather patterns, and combined results across cities within clusters using meta-smoothing.

**Results:**

There was substantial variation in the effects of the same temperature by month. Heat effects were larger in the spring and early summer and cold effects were larger in late fall. In addition, heat effects were larger in clusters where high temperatures were less common, and vice versa for cold effects.

**Conclusions:**

The effects of a given temperature on mortality vary spatially and temporally based on how unusual it is for that time and location. This suggests changes in variability of temperature may be more important for health as climate changes than changes of mean temperature. More emphasis should be placed on warnings targeted to early heat/cold temperature for the season or month rather than focusing only on the extremes.

**Electronic supplementary material:**

The online version of this article (doi:10.1186/1476-069X-13-89) contains supplementary material, which is available to authorized users.

## Background

The effects of temperature on public health are comprehensive and ubiquitous. Meanwhile, climate change is shifting the distribution of daily temperature upward, and may be increasing episodes of unseasonal temperature [1].

Many studies have attempted to understand how extreme temperature affects human health and mortality [2–6]. Generally, those approaches focused on dose-response relationships over an entire year. Other studies have suggested that temperature effects vary geographically with different threshold temperatures due to acclimatization to local weather [5, 7, 8]. This raises the question of whether temporal acclimatization to temperature matters as well as spatial acclimatization. That is, does the dose-response vary by time of the year?

There have also been some studies implying that timing of exposure to excessive heat matters for the magnitude of the adverse health outcome [9, 10]. They have found that early exposure to a heat wave has more impact than the same event later. However, those studies focused only on extreme events, early heat waves were not generally comparable in terms of the intensity and duration to later ones, and the definition of timing was descriptive. In this study, we investigated in a systematic way the effect of timing of exposure to both warm and cold temperatures treated as continuous predictors. Specifically, we examined the dose-response relationship separately in each city in each month, using sufficient years (1973-2006) to ensure stability of the estimates.

To further stabilize results we started with 148 US cities, and clustered them by similarity in seasonal mean and variance of temperature to obtain clusters of cities with similar weather. Results from cities belonging to the same cluster were combined to obtain a more robust estimate of how temperature effect varies by month, and the resulting exposure-response curves were compared among clusters. We also examined how the dose response curves varied by cluster, and the effect of timing by cluster.

## Methods

### Data

We obtained the data from 211 cities with complete mortality and weather variables for the study. In most cases, a city was contained by a single county. However, we used multiple counties where the city’s population extends beyond the boundaries of one county.

Among those cities, we restricted our analysis to cities with a daily average of 5 deaths per day or more for statistical robustness. As a result, we ended up with 148 cities.

Meteorological data were downloaded from the National Oceanic and Atmospheric Administration (NOAA) website and measured by airport weather stations. Since the data are from the airport weather stations, the measurements included visibility in meters as well as daily mean temperature, wind speed, sea level pressure, and dew point. Therefore, relative humidity was calculated with the following formula:


where T_a_ and T_d_ denote air temperature and dew point temperature, respectively [11].

Among weather monitoring stations, the closest one in distance was assigned to each city for ambient temperature and relative humidity. Since the weather stations were located in airports, the difference in altitude didn’t play a role. In case a monitor has missing data, we used the values of the nearest monitor within 60 kilometers. To remove erroneous readings without deleting true extreme events, temperatures out of the 8 standard deviation range were eliminated.

Daily mortality data, including the number of deaths for each day and cause of death, were obtained from the National Center for Health Statistics (NCHS), from the year 1973 through 2006 [4, 12]. We used deaths from any natural cause except for accidental causes (ICD-code 10th revision: V01-Y98, ICD-code 9th revision: 1-799), of persons who resided within the city where they died.

### Statistical analyses

Considering the huge variations in the climate of the United States, we categorized the 148 cities into 8 statistical clusters by seasonal temperatures and their seasonal variances. By doing this, we aimed to maximize the similarity within the cluster and dissimilarity between clusters at the same time. Specifically, we employed an agglomerative hierarchical approach where, we started by defining each data point to be a cluster and then combined existing clusters at each step through the single linkage method. PROC CLUSTER in SAS 9.2 (Copyright © 2012 SAS Institute Inc., SAS Campus Drive, Cary, North Carolina 27513, USA) was implemented based on the mean and standard deviation of the temperature for four seasons in each city.

The statistical analysis consists of two phases. In the first stage, separate daily Poisson time-series analyses were fit for each city and month of the year to evaluate the effect of temperature on mortality. Because we had 34 years of data for each month, we had sufficient power to estimate these effects. The effect of heat seems to primarily manifest within a day, whereas the effect of cold temperatures is spread out over more days. To accommodate this we fit two temperature variables, temperature on the day of death (lag 0), and the average temperature for the five previous days (lags 1-5). For consistency, temperatures were centered to 18°C. Since the association of temperature with mortality can be nonlinear, we used a penalized spline to estimate it. The model also controlled for the time trend of mortality and temperature over the 34 years by adding a linear term on the sequence of days. To check the collinearity between the lag 0 lag 1-5, the correlation coefficients were calculated. Day of week was also controlled. Specifically, we assumed:


where λ denotes the expected number of deaths on day *t* for city *i* in month *j*; Time_*i*_ is the sequence of days which counts within month and also increments with the calendar year in city *i*; TMP0 is the ambient temperature in Celsius on the same day of death in city *i*; s is the penalized spline function for the temperature effects, estimated with cubic regression splines with 10 knots; TMP15 is the moving average of 1-5 previous days from the death day; RH is the relative humidity; DOW is the indicator variable for day of week on day *t*. We assumed a quasi-Poisson distribution for λ to account for any over-dispersion.

In the second stage, we combined the curves from the previous model into a curve representing each month for each cluster. Doing this by cluster assured that the overlap in temperature range between cities was large, and that the dose-response curves were similar. Since the splines in the city specific models choose knot points based on the city specific distribution of temperature, a meta-analysis of the spline coefficients is not possible. To avoid this problem, we used meta-smoothing, a method introduced by Schwartz and Zanobetti to incorporate varying smooth curves into one overall curve [13]. It is based on the idea that predicted curves can be represented by using their predicted values for a dense range of points. Using the predicted values at those points, and their variance, we can do a point-wise meta-analysis.

In this study, we estimated predictions (and their confidence intervals) for each city/month for each 2°C interval. Next, we applied random effects meta-analyses for each temperature. Finally, by connecting the points, meta-curves were completed. We confined the meta-smoothing to the 99.9^th^ percentile temperature range to avoid extreme values with only one city contributing to the estimate. In the subgroup analysis, mortality due to respiratory disease was examined.

Humidity is a key factor for regulating the body temperature since it modifies the evaporation of sweat in hot weather. As a sensitivity analysis to examine the effect of relative humidity control, we reran the model without the relative humidity term.

Temperature effects may also be confounded by air pollution effects such as PM_10_ or PM_2.5_. Since these were never measured in some cities, and only in later years in others, we analyzed visibility instead as a surrogate for particles. Horizontal visibility is a sensitive indicator of fine particle concentrations [14]. And we repeated the meta-smoothing to compare the results with one from the original model.

## Results

**Figure 1:**
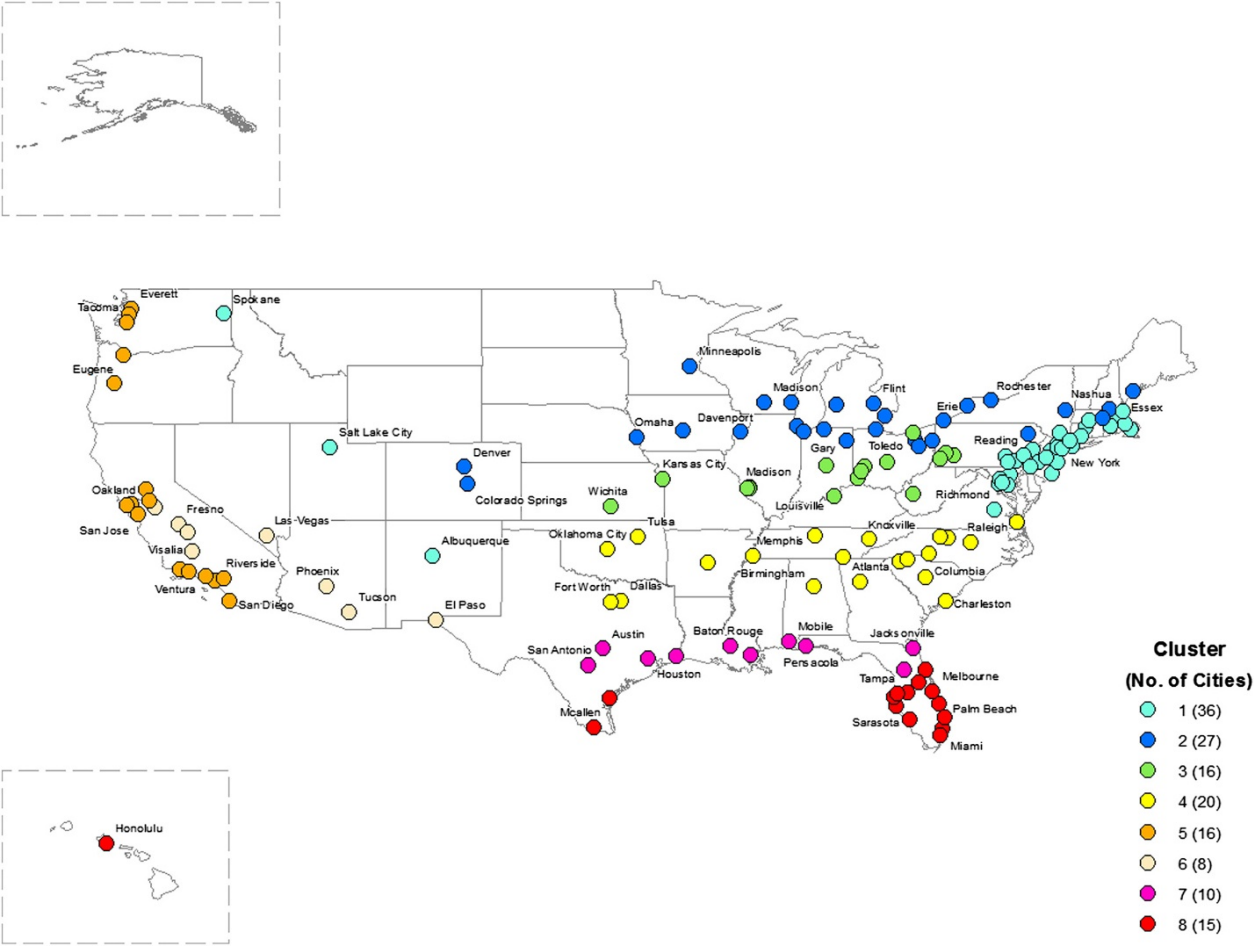
**Distribution of study area by cluster: Each color represents the cluster ID and the number of cities which belongs to each cluster is in the parentheses.**

38,005,616 deaths occurred in 148 cities between 1973 and 2006. Figure 1 and Table 1 show the location of 148 cities by cluster and the descriptive statistics of the temperature and mortality. The first cluster consists of 36 cities mainly located along the northern Atlantic coast area (New York City, Philadelphia, Boston, etc.) but also including some cities in the west (Spokane, Salt Lake City, and Albuquerque). The second cluster (27 cities) was the coldest region with cities such as Chicago, Detroit, and Minneapolis. The third cluster (16 cities), the secondly coldest area, had cities such as Cleveland, Pittsburgh, and St. Louis. Cluster 4 is comprised of 20 warm cities with mild winter temperature such as Atlanta, Charlotte, and Dallas. Cluster 5 contains 16 cities along the west coast (Los Angeles, San Francisco, and Seattle). The sixth cluster consists of 8 cities with very hot and dry weather such as Las Vegas and Phoenix. The seventh cluster is a hot and humid area including 10 cities such as New Orleans, Austin, Houston, etc. Lastly, the eighth cluster is made up of 15 tropical cities such as Miami and Honolulu.

**Table 1:** **Descriptive statistics of temperature and mortality by cluster**

Cluster	Season	Temperature (°C)		Relative humidity (%)		Daily death (Count)	
		Mean	S.D.*	Mean	S.D.	Mean	S.D.
1	Spring-Summer	16.83	8.00	63.55	16.84	22.63	33.08
	Fall-Winter	7.03	8.53	66.20	15.41	24.29	35.57
2	Spring-Summer	15.12	8.63	65.12	14.97	19.00	28.11
	Fall-Winter	4.13	9.56	69.76	14.33	20.22	29.88
3	Spring-Summer	18.04	8.16	65.77	13.43	17.29	13.67
	Fall-Winter	7.03	9.38	69.47	13.18	18.43	14.56
4	Spring-Summer	21.10	6.74	65.86	13.92	12.33	9.07
	Fall-Winter	11.58	8.14	66.82	15.52	13.20	9.70
5	Spring-Summer	16.79	4.93	66.56	13.83	29.16	33.50
	Fall-Winter	12.83	5.65	70.42	17.25	31.16	36.35
6	Spring-Summer	23.67	7.09	37.35	17.40	13.50	12.50
	Fall-Winter	14.76	7.36	52.66	21.91	14.51	13.40
7	Spring-Summer	23.93	5.01	70.99	12.20	13.47	11.58
	Fall-Winter	16.69	6.99	71.13	14.42	14.34	12.28
8	Spring-Summer	25.29	3.63	71.52	9.69	15.61	11.76
	Fall-Winter	21.02	5.44	73.13	10.87	16.39	12.22

*S.D. is the standard deviation.

**Figure 2:**
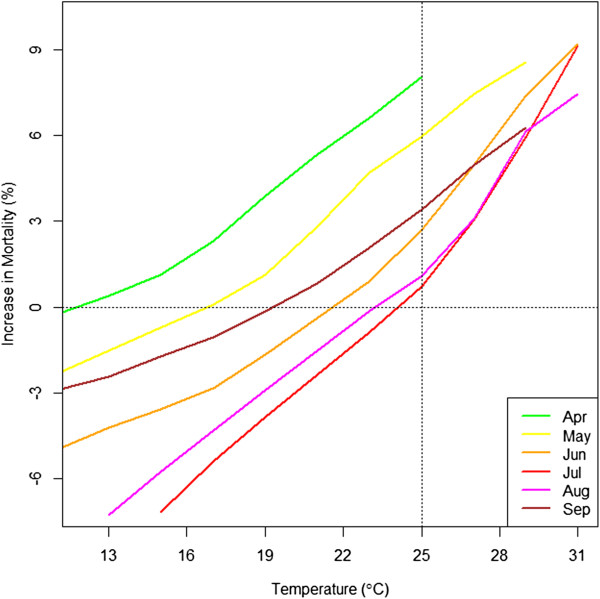
**Heat effects by month in cluster 1: The percent change in mortality in association with heat compared to the mortality at the mean temperature in corresponding month differs by month.**

**Figure 3:**
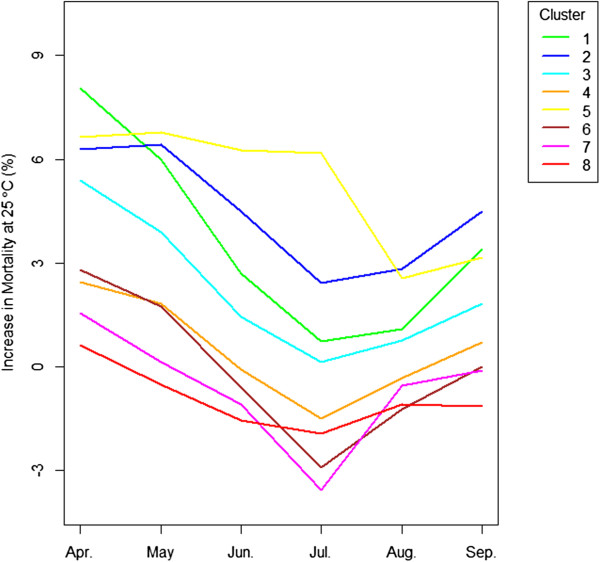
**Monthly trend of mortality at 25°C by cluster: The mortality response at 25°C compared to the mortality at the mean temperature in corresponding month and cluster shows the U-shape trajectories.**

Figure 2 shows the monthly effects of heat on mortality (i.e. lag 0 temperature) in cluster 1. We present the results from this cluster because it is the one of the most seasonal cluster and also takes the largest number of cities among clusters. Each curve represents a month from April to September and shows the percent increase in mortality at each temperature compared to the mortality at 18°C. The results clearly differ by month, with the same temperature having the largest effect on excess of mortality when it occurs in April, progressively lower relative impacts as summer develops, and increasing again in fall. Specifically, mortality increases by 8.69% at 25°C compared to 18°C in April, by 6.77% in May, and by only 2.98% in June, which shows the decrease in the increment of mortality. In July, the midst of summer, the increase in mortality at 25°C hits its minimum, which is 0.72%. It recovers in August to 1.23% and increases further to 3.51% until September. This pattern was consistently observed in other clusters as well except cluster 5 (results not shown). In Figure 3, the monthly trajectories of the increase in mortality at 25°C are shown by clusters. In almost every cluster, the increases in mortality at 25°C peak at April, and decrease until they hit bottom in July (or August in cluster 5). The effect then rebounds into the fall. Those V-shaped curves demonstrate that exposure timing defined by month played a significant role in the relationship between temperature and mortality. That is, at the same temperature, the excess mortality response differed depending on when people were exposed to it. Table 2 suggests the 95% confidence intervals for Figure 3. The confidence intervals for cluster 1 which has the greatest number of cities (36) don’t overlap implying that these trends are statistically significant. Due to the lack of number of cities, the confidence intervals from other clusters display a degree of overlaps. It also shows the amount of increase in mortality was not symmetric between the early season and the late season. It showed a smaller increase in mortality in September than in May. In addition, it illustrates the statistically substantial differences in the mortality effect by cluster.

**Table 2:** **Percent increase in mortality at 25°C by cluster and month**

	April	May	June	July	August	September
1	8.69 (7.16, 10.25)	6.77 (5.52, 8.04)	2.98 (2.57, 3.39)	0.72 (0.48, 0.96)	1.23 (0.90, 1.57)	3.51 (2.77, 4.26)
2	6.58 (4.63, 8.56)	6.19 (4.94, 7.46)	4.26 (3.53, 4.99)	2.20 (1.78, 2.62)	2.92 (2.29, 3.56)	4.74 (3.67, 5.81)
3	5.09 (3.21, 7.01)	3.60 (2.70, 4.51)	1.39 (0.99, 1.80)	-0.02 (-0.13, 0.09)	0.70 (0.46, 0.94)	2.13 (1.30, 2.97)
4	2.89 (1.68, 4.12)	1.87 (1.10, 2.65)	0.19 (0.04, 0.35)	-2.10 (-2.87, -1.33)	-0.82 (-1.16, -0.48)	0.82 (0.31, 1.33)
5	5.35 (1.34, 9.51)	6.40 (4.07, 8.79)	7.33 (4.89, 9.82)	6.52 (5.21, 7.85)	4.82 (2.99, 6.69)	5.93 (3.95, 7.95)
6	2.74 (1.03, 4.49)	0.88 (0.36, 1.39)	-1.31 (-2.06, -0.54)	-3.19 (-6.26, -0.02)	-2.14 (-3.57, -0.70)	-0.49 (-0.77, -0.21)
7	1.62 (0.38, 2.87)	0.39 (0.04, 0.75)	-1.54 (-2.25, -0.81)	-2.8 (-4.30, -1.26)	-0.74 (-2.06, 0.61)	-0.02 (-0.25, 0.20)
8	0.61 (0.11, 1.11)	-0.36 (-0.62, -0.10)	-2.22 (-3.12, -1.31)	-2.63 (-4.05, -1.20)	-2.30 (-3.73, -0.85)	-1.59 (-2.61, -0.56)

Estimate is percent increase in mortality at 25°C compared to mortality at 18°C.

Negative value means lower mortality than the reference temperature of 18°C.

() is 95% confidence interval.

**Figure 4:**
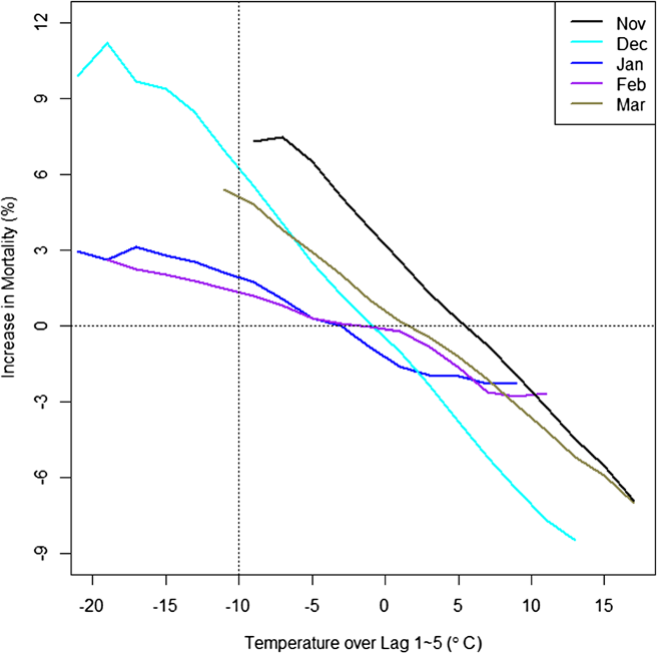
**Cold effects by month in cluster 2: The percent change in mortality in association with cold compared to the mortality at the mean temperature in corresponding month differs by month.**

Figure 4 shows a similar pattern during the cold months. The effects of cold temperatures (lag 1-5) are the smallest in January and February and larger in December, November, and March. We present the results from cluster 2 since it has the next largest number of cities and to show the results from other than cluster. We observed that the early season effect occurred even in the coldest region, cluster 2. The increase in mortality at -10°C is much higher in December compared to other months in the middle of winter at the same temperature. Again, there seemed to be an asymmetry in effects over the cold season, as the effect in March was lower than the effect of the same temperature in November. For reference, correlation between lag 0 and lag 1-5 was the average of 0.53.

**Figure 5:**
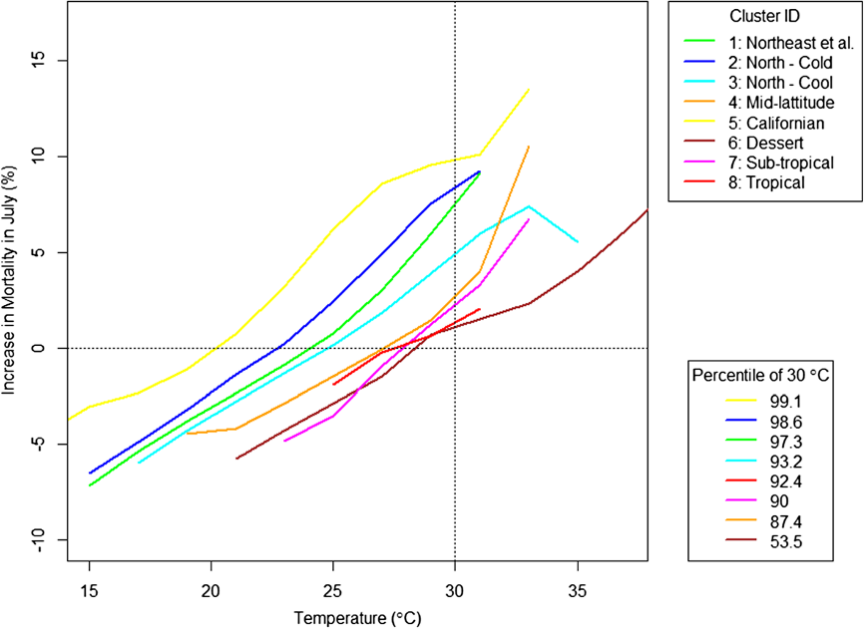
**Heat effects in July by cluster: The response to the heat effects differs by regions and it depends on the relative scale rather than the absolute temperature.**

We also found the geographic differences in the response to temperature, when investigated by cluster. Figure 5 shows how heat effects differ by region in July. At 30°C, cluster 5 shows the highest mortality, followed by clusters 2, 1, 3, which are located in cold regions. Mortality at that temperature was lowest in the desert and tropical clusters (6 and 8). Looking at the percentile of temperature that corresponds to 30°C tells the same story. In cluster 5, 30°C is the 99.1^th^ percentile, and is associated with the largest percentage increase in mortality amongst the clusters. The same temperature ranks as the 98.6 percentile in the second cluster leading to the second highest increase in mortality and so forth.

**Figure 6:**
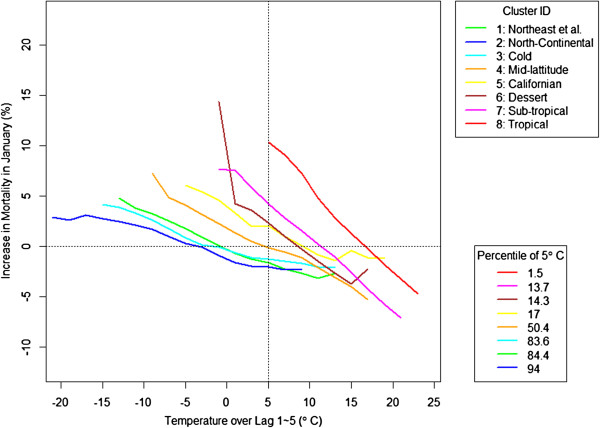
**Cold effects in January by cluster: The response to the cold effects differs by regions and it depends on the relative scale rather than the absolute temperature.**

Regional differences in mortality were also observed for the cold effect (shown in Figure 6) and the difference was more drastic than for the heat effects. As the region moves from cold to hot, the increase in mortality at a given temperature increases rapidly. While for the heat effects, the dose-response curves were generally parallel, with similar slopes but different intercepts, for cold temperature, the slopes change substantially between regions. As with the heat effect, the percentile of temperature was generally identical to the rank of increase in mortality by clusters. Cluster 8, the tropical cluster, was the most vulnerable region to cold.

In the subgroup analysis, mortality due to respiratory disease showed the greatest difference between December and February (see Additional file 1). In February, the mortality even decreases.

**Figure 7:**
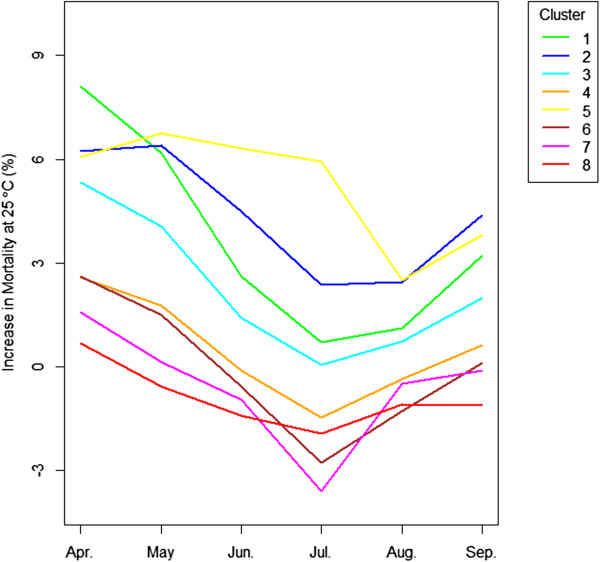
**Sensitivity analysis on visibility - monthly trends of mortality at 25°C.**

The sensitivity analysis controlling for visibility had little effect. Rather, the addition of the visibility variable has increased the effect estimates slightly (Table 3 and Figure 7).

**Table 3:** **Sensitivity analysis – percent increase in mortality at 25°C by cluster and month**

	April	May	June	July	August	September
1	9.04 (7.42, 10.68)	7.02 (5.60, 8.47)	2.79 (2.27, 3.32)	0.66 (0.41, 0.91)	1.25 (0.92, 1.59)	3.45 (2.69, 4.21)
2	6.48 (4.56, 8.43)	6.00 (4.78, 7.24)	4.08 (3.25, 4.92)	1.98 (1.53, 2.44)	2.63 (1.94, 3.31)	4.38 (3.19, 5.58)
3	4.88 (2.98, 6.82)	3.72 (2.80, 4.65)	1.50 (1.06, 1.94)	-0.03 (-0.15, 0.09)	0.61 (0.33, 0.88)	2.08 (1.15, 3.02)
4	2.93 (1.75, 4.13)	1.65 (0.80, 2.52)	0.22 (0.05, 0.38)	-1.93 (-2.70, -1.15)	-0.74 (-1.14, -0.35)	0.77 (0.27, 1.28)
5	5.58 (1.22, 10.14)	6.69 (4.41, 9.02)	7.24 (4.74, 9.79)	6.29 (4.90, 7.69)	4.33 (2.80, 5.88)	6.18 (4.17, 8.23)
6	2.82 (0.60, 5.10)	0.64 (0.06, 1.22)	-1.43 (-2.21, -0.65)	-3.91 (-6.76, -0.97)	-2.40 (-3.84, -0.94)	-0.51 (-0.78, -0.24)
7	1.64 (0.13, 3.18)	0.38 (0.01, 0.74)	-1.48 (-2.25, -0.70)	-2.74 (-4.25, -1.20)	-0.64 (-1.98, 0.72)	-0.04 (-0.27, 0.19)
8	0.65 (0.09, 1.21)	-0.38 (-0.62, -0.14)	-2.05 (-2.95, -1.14)	-2.56 (-3.96, -1.15)	-2.40 (-3.90, -0.88)	-1.42 (-2.30, -0.52)

Sensitivity analysis for the addition of visibility.

Estimate is percent increase in mortality at 25°C compared to mortality at the mean of each cluster and month.

Negative value means lower mortality than the reference temperature of 18°C.

() is 95% confidence interval.

The deletion of relative humidity from the model produced similar results although it decreased both estimates for heat and cold (Additional file 1). By cluster, the removal of the relative humidity affected cluster 5 (California), where estimate for April increased substantially (Additional file 1). Compared to the main model, the increase in mortality is less until June; however after that, the estimates were greater than the main model (Additional file 1).

## Discussion

In this study, we demonstrated that the response to a given temperature depends on the month it occurs in, and that the response varies across clusters defined by similar temperature and humidity patterns. Furthermore, it appears that the earlier people are exposed to extreme temperatures for the season the higher the increase in mortality. This finding within each cluster is paralleled by the finding across cluster that at a given temperature in a given month (e.g. 30°C in July) the mortality response by cluster depends on the degree to which the temperature is unusual. This finding is consistent with other studies that found higher effects of early season exposure [9, 10, 14, 15]. This phenomenon might be explained by mortality displacement, where the vulnerable population dies off in the first heat wave. However, that does not explain phenomena in Figure 2 showing the constant pattern of monthly effects because if the vulnerable population dies off in earlier season such as April, the deceasing pattern of mortality will stop at the next month such as May or June at the same temperature of 25°C. It also does not explain the bounce back of the increase in mortality in Figure 3. Again, if the vulnerable population dies off, the mortality will keep decreasing until September, not recover at the same temperature. Moreover, mortality displacement [16] after the depletion of susceptible persons is usually observed in a period of a week [10]. Therefore, a monthly difference may not be explainable solely by the harvesting effect. This still applies even if the depletion of the susceptible takes longer than a week based on the phenomenon in Figure 2 and Figure 3.

Another plausible explanation would be the temporal acclimation over the course of a season. This can be due to physiological adaptation or behavioral change. Physiological acclimation develops over the course of seasons. For example, as summer progresses, the sweat glands expand and cardiac output increases to sweat, and the concentration of sodium in the same amount of sweat becomes diluted [17]. Exposure to heat before such acclimation completes can be more hazardous, and the risk of illness is greatest during the first week of unusual heat [18, 19]. Meanwhile, physiological adaptation doesn’t last long and can decay within a few days or weeks after removal from heat [20–22]. This may explain the bounce back of mortality in Figure 3. The non-symmetry in the amount of the increase in mortality between the early season and the late season may be explained by the remaining effects of acclimation. Behavioral adaptation such as wearing more clothes or the use of air conditioners is another key factor for lowering mortality. However, early exposure to heat/cold might occur before behavioral adaptation. The public may neglect to prepare themselves for early heat or cold, compared to those in the middle of season. The public should be notified that 25°C in May can be as harmful to health as 29°C in July.

For cold, there were more cumulative effects defined by lag 1 through lag 5. This could be because mortality due to cold is indirect, through illnesses such as pneumonia and influenza [23]. Mortality due to respiratory causes also showed a huge difference between the induction of the season (December) and the middle of the season (February). And it appears that the retention of acclimation lasts longer for cold than heat, considering that February showed the lowest mortality whereas July had the lowest mortality effect in summer.

Our findings suggest that if the effects of temperature are highly time dependent (i.e., differ by specific month), investigating temperature by season or only by year effectively averages over diverse months. Therefore, summing up temperature effects and ignoring the timing would dilute the effects of ambient temperature, reducing the estimated change in mortality per unit change in temperature.

We also found that spatial differences in the temperature effect on mortality. Cluster 6, characterized by a hot and dry climate, showed the strongest resistance to the heat. The first possible hypothesis is that the low relative humidity in those dry areas contributed to this high resistance to the heat. It appears that heat acclimation remains longer for dry heat compared to humid heat [24]. It could also be due to the prevalence of air conditioning. Lastly, compared to cluster 8, which is a tropical region, a wider range of heat temperature may have provoked the adaptation to the variability of temperature. For cold, the regional difference was greater than the heat effects. This suggests that human adapt better to cold than to heat.

Removal of the relative humidity from the model made estimates for early summer decrease but increases estimates for late season and winter. This might imply the adaptation is also going on for humidity as well as temperature.

Our results were not confounded by visibility, which is a surrogate measurement of particulate matter such as PM_10_ and PM_2.5_. Rather, the addition of visibility increased the model estimates for temperature. Other studies also state that the relationship between temperature-mortality is robust to air pollution control [4, 8].

The main limitation of the study is the use of ambient temperature as a surrogate for personal exposure. Personal exposure to ambient temperature is modified by adaptive mechanisms such as use of air conditioning. Actual outdoor temperature also can be altered from the airport monitoring stations due to the distance from the monitors and the difference in topography and elevation. Nevertheless, our results are conservative, because the measurement error is non-differential to the outcome. With an ongoing attempt to precisely predict temperature [25], exposure measurements will be improved.

Since cardiovascular stability is critical in heat acclimation and is also affected by cold, compromises in this ability will pose more severe burdens on the elderly and the ill. In future studies, subgroup analyses for these populations will reveal more about the impact of monthly temperature anomalies.

Our study has many policy implications. The monthly effects of temperature suggest that more warnings should be given to the public for hot and cold events early in the season as they occur before acclimation has developed. The media and many studies are interested in peak temperatures such as 40°C in the middle of summer. Yet our findings indicate that the impact of early events of less extreme temperature may be greater. Also, warnings could be provided based on a relative scale, such as a percentile, as well as the absolute scale of temperature. In July, 25°C is merely the 49^th^ percentile, whereas the same temperature is the 86^th^ percentile in May, and it poses more harm to the public in the earlier season. To the extent that climate change increases the occurrence of early season warm or cold days, this may be an important health consequence of such changes.

To our knowledge, this is the first study to examine the dependence on month of the effects of temperature on mortality. Timing of exposure to extreme temperature should be given more attention in terms of acclimation. Early heat and cold pose a higher risk, as people are not prepared for them. Furthermore, due to climate change, it is projected that unseasonal days will be increasing and arriving earlier. It is necessary to prepare for these hazards.

## Conclusions

The effects of a given temperature on mortality vary spatially and temporally based on how unusual it is for that time and location. This suggests changes in variability of temperature may be more important for health as climate changes than changes of mean temperature. More emphasis should be placed on warnings targeted to early heat/cold temperature for the season or month rather than focusing only on the extremes.

## Electronic supplementary material

Additional file 1: Figure S1: Mortality due to Respiratory disease in Cluster 1. **Figure S2.** Comparison between Main Model and RH model in Cluster 1. **Figure S3.** Monthly Trend of Mortality at 25°C by Cluster (RH Deletion). **Table S1.** Monthly Trend of Mortality at 25 C in Cluster 1 (RH Deletion). (DOCX 114 KB)
