# Supplementary material for: Acclimatization across space and time in the effects of temperature on mortality: a time-series analysis
Source: Environ Health. 2014 Oct 28;13:89. doi: 10.1186/1476-069X-13-89 (PMC4271464; doi:10.1186/1476-069X-13-89)
Supplement: Supplementary file 1 — Additional file 1: Figure S1: Mortality due to Respiratory disease in Cluster 1. Figure S2. Comparison between Main Model and RH model in Cluster 1. Figure S3. Monthly Trend of Mortality at 25°C by Cluster (RH Deletion). Table S1. Monthly Trend of Mortality at 25 C in Cluster 1 (RH Deletion). (DOCX 114 KB) [file 12940_2014_807_MOESM1_ESM.docx]

## Additional file 1


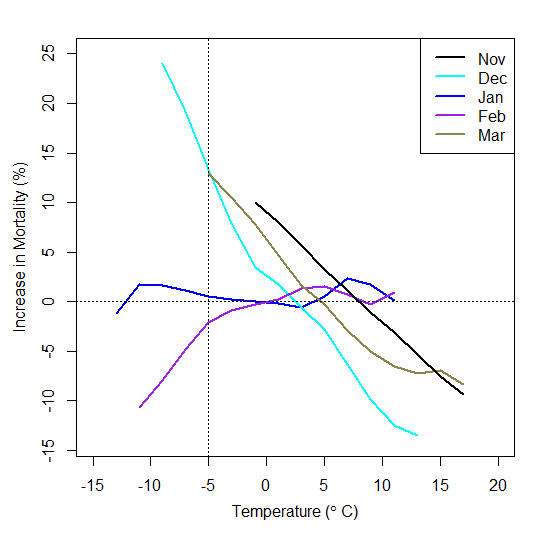


Figure S1. Mortality due to Respiratory disease in Cluster 1


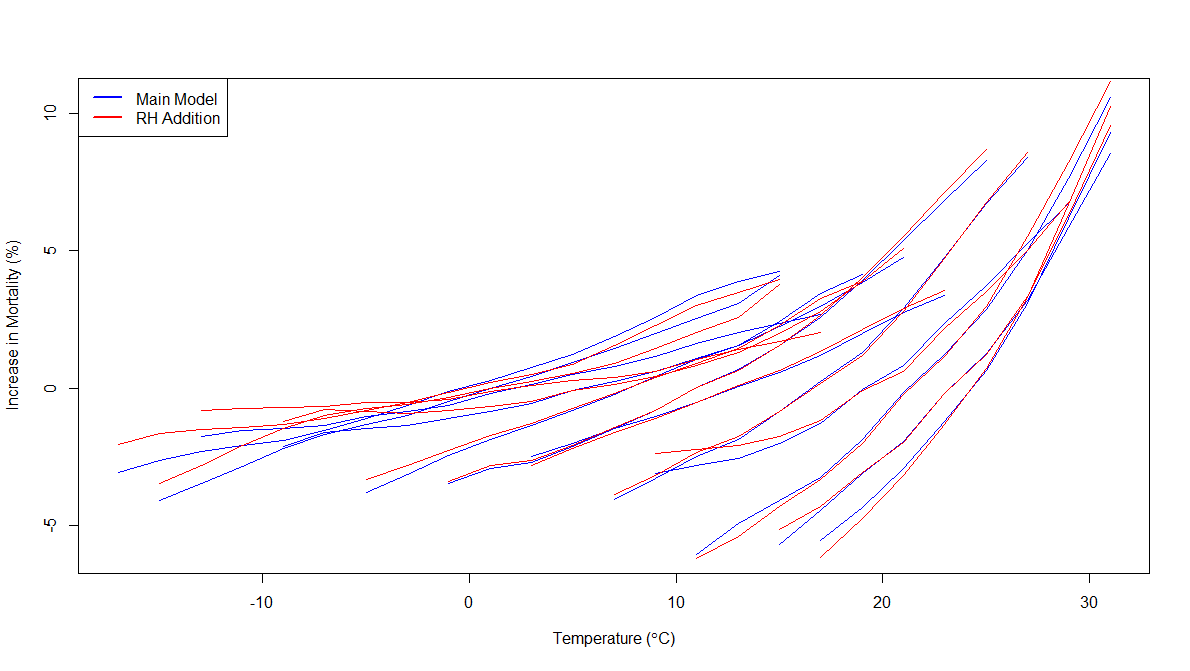


Figure S2. Comparison between Main Model and RH model in Cluster 1


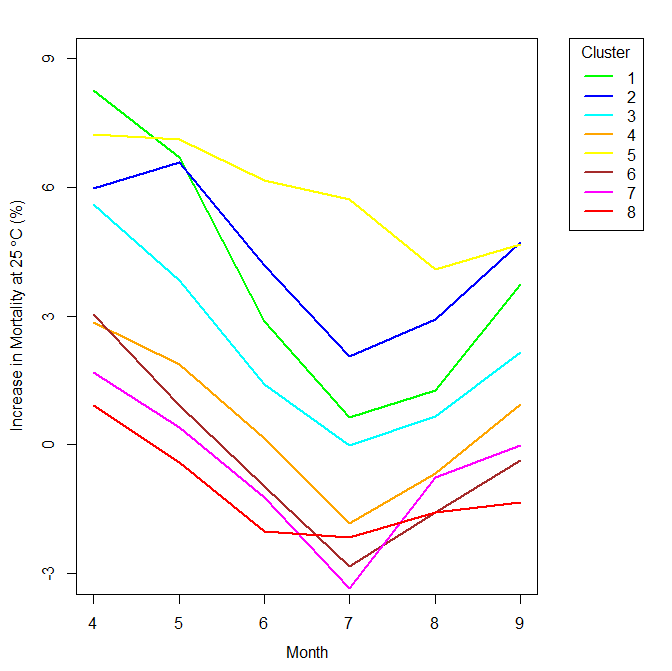


Figure S3. Monthly Trend of Mortality at 25 °C by Cluster (RH Deletion)

Table S1. Monthly Trend of Mortality at 25 C in Cluster 1 (RH Deletion)

|  | April | May | June | July | August | September |
| --- | --- | --- | --- | --- | --- | --- |
| 1 | 8.27 (6.82, 9.73) | 6.71 (5.58, 7.85) | 2.87 (2.37, 3.37) | 0.64 (0.45, 0.84) | 1.27 (0.99, 1.54) | 3.74 (3.04, 4.43) |
| 2 | 5.97 (4.24, 7.73) | 6.59 (5.55, 7.65) | 4.19 (3.46, 4.93) | 2.06 (1.71, 2.41) | 2.93 (2.41, 3.46) | 4.71 (3.73, 5.71) |
| 3 | 5.61 (3.45, 7.83) | 3.84 (2.91, 4.78) | 1.4 (1.01, 1.79) | -0.02 (-0.13, 0.09) | 0.67 (0.44, 0.89) | 2.15 (1.31, 2.99) |
| 4 | 2.85 (1.65, 4.06) | 1.88 (1.1, 2.65) | 0.16 (0.02, 0.31) | -1.84 (-2.47, -1.2) | -0.66 (-0.96, -0.36) | 0.95 (0.5, 1.4) |
| 5 | 7.25 (3.88, 10.72) | 7.12 (4.8, 9.5) | 6.17 (4.07, 8.31) | 5.73 (4.4, 7.08) | 4.09 (3.23, 4.96) | 4.67 (3.3, 6.05) |
| 6 | 3.05 (1.48, 4.66) | 0.92 (0.38, 1.46) | -0.98 (-1.65, -0.31) | -2.83 (-4.83, -0.78) | -1.57 (-2.64, -0.49) | -0.37 (-0.79, 0.06) |
| 7 | 1.68 (0.24, 3.14) | 0.41 (0.06, 0.76) | -1.22 (-1.91, -0.53) | -3.35 (-4.73, -1.95) | -0.75 (-1.9, 0.42) | -0.01 (-0.25, 0.23) |
| 8 | 0.93 (0.48, 1.38) | -0.4 (-0.67, -0.13) | -2.02 (-2.82, -1.21) | -2.16 (-3.25, -1.07) | -1.57 (-2.68, -0.46) | -1.34 (-2.07, -0.6) |

Estimate is percent increase in mortality

() is 95% confidence interval
